# How a Novel Approach of Allergy Call Center Improved the Management of the Anti-COVID Vaccination Campaign in Piedmont: Italy

**DOI:** 10.1007/s44197-024-00309-2

**Published:** 2024-10-14

**Authors:** Iuliana Badiu, Stefania Nicola, Nicolò Rashidy, Stefano Della Mura, Daniele Tarrini, Virginia Bernardi, Mara Gallicchio, Irene Ridolfi, Elena Saracco, Erika Montabone, Marina Mazzola, Luca Lo Sardo, Giada Geronazzo, Ludovica Comola, Antonietta Apricena, Ilaria Vitali, Anna Quinternetto, Lucrezia Alessi, Federico Meli, Marzia Boem, Marcelo Teocchi, Salvatore Schinocca, Maria Carmen Rita Azzolina, Federica Corradi, Simone Negrini, Giovanni Rolla, Richard Borrelli, Luisa Brussino

**Affiliations:** 1 Department of Medical Sciences, Immunology and Allergy Unit, University of Turin, Mauriziano Hospital, 10128 Turin, Italy; 2 Health Management, Mauriziano Hospital, 10128 Turin, Italy; 3 Department of Internal Medicine, Clinical Immunology and Translational Medicine Unit, University of Genoa and IRCCS Ospedale Policlinico San Martino, Viale Benedetto XV, 6, 16132 Genoa, Italy

**Keywords:** Allergy call center, Vaccination campaign, COVID-19, SARS-CoV-2 vaccine, Allergy, Allergy risk, Consultations

## Abstract

**Objective:**

The anti-COVID vaccination campaign has led to a significant increase in the demand for allergology consultations in patients considered at risk of reaction to anti-COVID-19 vaccines. This study aims to describe the experience of the vaccination campaign held in Piedmont (Italy) which developed a new service of Allergy Call Center (ACC) thus providing for the screening and management of allergy high-risk patients during pandemic.

**Study Design:**

A retrospective analysis was performed on all patients considered at high risk for the development of allergic reactions who were referred by the Immunology and Allergy Unit of Azienda Ospedaliera Ordine Mauriziano in Turin, Italy, between December 2020 and December 2022 and also on ACC consultations.

**Methods:**

During the COVID-19 pandemic, Piedmont Region instituted the ACC, active from May 10th, 2021 to December 31st 2022, to allow vaccinating doctors to require a telephonic consultation for patients who were considered at high risk for the development of allergic reactions. If further diagnostic evaluations were required, the ACC scheduled a visit with a Consultant of the Unit to better assess the clinical situation of the patient. Furthermore, patients referred by General Practitioners, Occupational Doctors and other consultants were also evaluated by the Unit when required.

**Results:**

During the operational period the ACC received a total of 15,865 calls and referred only 336 patients to the unit (27.4% of the total referrals), while General Practitioners referred 499 patients (40.8%), Occupational Doctors referred 61 patients (4.9%), and other consultants referred 326 patients (26.6%).

**Conclusions:**

Evaluation and management of a large volume of requests seemed to be facilitated by a proactive framework for screening patients at high risk for allergic reactions as the ones referred by our ACC. This approach led to a prominent decrease in allergological visits to our tertiary care Centre, reducing the waiting times and providing additional support for both patients and healthcare providers, thus allowing the vaccinations to be more easily handled.

**Supplementary Information:**

The online version contains supplementary material available at 10.1007/s44197-024-00309-2.

## Introduction

The COVID-19 pandemic has, to date, resulted in approximately 774 million confirmed cases and caused nearly 6.9 million deaths worldwide [1]. Vaccination is considered the most valua-ble tool to control the pandemic of COVID-19; however, since their introduction, the success of the vaccination campaign relied mostly on the population’s willingness to get vaccinated. Con-cerns about vaccine safety and efficacy, coupled with widespread misinformation, have gener-ated a phase of hesitancy which left both the population and healthcare professionals with un-certainties and unmet needs. A recent Italian meta-analysis reported a significant percentage of hesitation among healthcare workers during the vaccination campaign. The main reasons for vaccine reluctance were the lack of information about vaccination, the uncertainty concerning its safety profile and the relative fear of adverse events.

The risk of anaphylaxis after vaccination with Comirnaty has been reported to be higher than other non-covid vaccinations [3–5] and the allergic sensitization was attributed to both the vac-cine itself and its excipients [6–9]. Specific recommendations suggested to screen patients with a history of allergic reactions before immunizing them with COVID-19 vaccines and also to evalu-ate patients who referred adverse reactions to a prior SARS-CoV-2 vaccine [10, 11]. This condi-tion has led to a notable increase in the request for allergy evaluations before vaccination, in subjects reporting adverse reactions to drugs containing Polyethylene glycol (PEG) or Polysorb-ate 80 (PS-80), as well as for those with suspected hypersensitivity reactions to a prior anti-COVID-19 vaccine [10, 11].

In Italy, the COVID-19 vaccination campaign started on 27 December 2020; as of April 2021, vaccination became mandatory for all healthcare workers (HCWs) as it was for individuals aged 50 and above from the beginning of January 2022. Piedmont (one of the biggest regions in the North-West of Italy) tried to simplify the process of allergic evaluations by setting an Allergy Call Center (ACC), managed by allergy specialists; this service was provided to all vaccinating doctors within the D.I.R.M.E.I. (Dipartimento Interaziendale funzionale a valenza Regionale Malattie ed Emergenze Infettive—Interdepartmental Service for the Assessment and Evaluation of Allergic Reactions) as it was inten ded to help physicians working at vaccination centres and general practitioners involved in the vaccination campaign. It remained active throughout the entire campaign period, namely till December 2022. The ACC followed the operative protocol for the assessment of the Allergological risk to SARS-CoV-2 vaccines developed by the Allergol-ogy and Immunology Unit of Azienda Ospedaliera Ordine Mauriziano in Turin [12this protocol was based on Italian and International guidelines [10, 11] and it aimed to identify high-risk pa-tients who required specific assessments, and to refer them when needed for additional evalua-tions.

At the very beginning of the campaign, our Unit also evaluated patients sent by occupational medicine doctors, consultants, and general practitioners.

Considering the experience of previous large vaccination campaigns, such as the anti-polio campaigns, literature studies underlined the usefulness of call center to increase vaccination adherence in the population [13, 14].

The aim of such services varied across different studies, from reminding patients of their ap-pointments to rescheduling activity in case of a missed vaccination appointment so as to meet the patients’ needs and enhance their compliance [14]. With the advent of the SARS-CoV-2 pan-demic, call centers have become a fundamental tool in attempting to break the spread of the in-fection through contact tracing and patient isolation. However, in almost all cases, the service was intended for the general population and not for healthcare providers.

In 2022 an Israeli study reported the first case of a call center service specifically established for healthcare providers with the purpose of supporting them in the general setting of COVID-19 management and in applying the national public health guidelines [15]; however, this service was not meant for being used as a specific tool for allergy evaluation.

A universal vaccination strategy was associated with a favourable cost–benefit ratio in a Health Technology Assessment (HTA) policy [16] as the risk-stratified approach to vaccination showed to be the most effective. As of today, no data regarding specific call center services for healthcare practitioners specifically intended to help them rule out suspected reactions in high-risk patients have been reported in the literature.

With this study, we aim to share the experience of the vaccination campaign held in Turin (Piedmont, Italy), which implemented a novel approach of the allergy telephone consultations as it allowed medical doctors with expertise in Immunology to work as a first line of filtration to better assist general practitioners and vaccination hubs.

## Materials and Methods

A retrospective analysis was performed on two sets of data: the logs of all the phone calls re-ceived by the Allergy Call Center (ACC) over several months, and the information of all the pa-tients referred by the Immunology and Allergy Unit of Azienda Ospedaliera Ordine Mauriziano in Turin, Italy, between December 2020 and December 2022. The data were evaluated to as-sess the risk of vaccination prior to administering the vaccine or in cases of suspected hypersen-sitivity reactions to vaccination.

Prior medical history, gender, and age of each patient were collected so that potential responses could be evaluated, and risk could be stratified. Patients who were already in care for allergy, anaphylaxis, asthma, or systemic mastocytosis were excluded from the study.

Patients were referred to the vaccination campaign by occupational physicians, general practi-tioners, or other in-hospital consultants. The ACC was established and maintained operational from the onset of the vaccination campaign (May 10th, 2021) until December 31st 2022.

Based on their medical histories, the ACC identified patients at moderate to high risk and re-ferred them to our Unit via a specific channel for consultation, thereby reducing the time re-quired to schedule the appointment.

Following our internal guidelines and the recommendations set by the Italians Societies for Al-lergies, Asthma and Clinical Immunology (AAIITO/SIAAIC) and the European Association for Al-lergy and Clinical Immunology (EAACI), individuals were classified as low, moderate, or high-risk patients based on their medical history [10–12].

Patients with low-medium risk were advised to receive vaccination in a standard setting with an observation time of 15, 30 or 60 min, depending on whether they had a history of anaphy-laxis.

Allergy tests for excipients were carried out in high-risk patients with a record of immediate al-lergic reaction to drugs containing PEG and/or PS80 if no other drugs containing them were administered. Patients with suspected immediate hypersensitivity reactions to anti-SARS-CoV-2 vaccines were tested as well. The procedures were performed by trained medical-nursing staff as per current guidelines [10, 11].

Patients who obtained negative results from allergological tests were considered eligible to re-ceive vaccinations. When immediate skin tests for excipients yielded a positive result, the pa-tient was considered ineligible to receive a vaccine containing the excipient for which they had developed a sensitization. The use of an alternative vaccine was advised if the alternative excip-ient reported negative to the test, as recommended by the guidelines [10].

Every patient was given comprehensive information concerning their eligibility for vaccination, the specific vaccine to be administered, the recommended observation period following vac-cination,

and, if required, the administration process in a secure hospital setting.

All methods, including the consent, were carried out according to relevant guidelines and the Declaration of Helsinki of 1964 and following legislative regulations.

The information was gathered and analyzed with the help of Excel (Microsoft Office, Build 14332.20615, Windows 11).

## Results

**Fig. 1:**
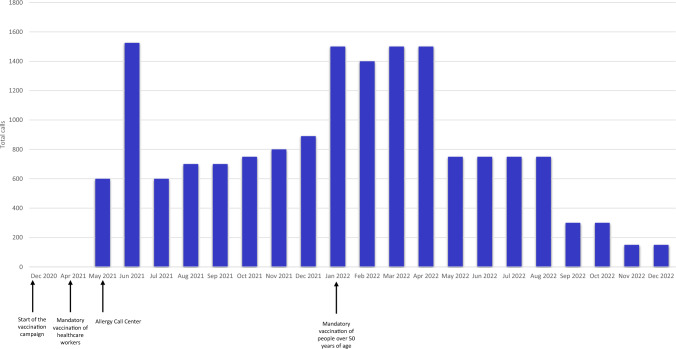
Number and distribution of the calls to the DIRMEI center between May 2021 and December 2022

The Allergy Call Center received a total of 15,865 calls during its operational period and testing phase. Figure 1 shows the monthly phone call report of the Allergy Call Center, with an estimat-ed daily mean of 27 consultations. In June 2021 and January-April 2022, the ACC peaked at 1500 phone calls per day. This increase occurred shortly after the service was launched as the vac-cines became mandatory for the Italian population.

**Fig. 2:**
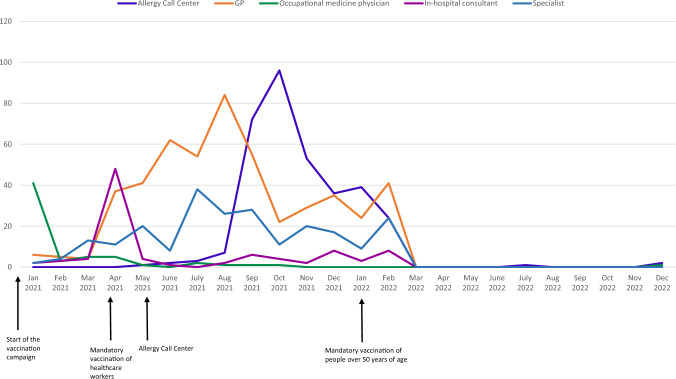
Monthly distribution of patients evaluated, categorized by the type of requesting doctor

The Allergy Call Center referred 336 patients, who accounted for 27.4% of the total referrals. General Practitioners referred 499 patients (40.8%), whilst Internal medicine consultants re-ferred 95 patients (7.7%). Occupational medicine physicians referred 61 patients, making up 4.9% of the referrals, while other medical specialists referred 231 patients, accounting for 18.9% of the total. Figure 2 illustrates the monthly distribution of patients evaluated, catego-rized by the type of requesting doctor.

A total of 1222 patients were evaluated, (mean age 52, F:M 4:1). Among them, 775 individuals (61.7%) were assessed in their first allergological evaluation. Out of a total of 978 individuals, the consultation was advised in 80% of cases since a previous negative reaction to a drug con-taining the excipients could be verified. Among this cohort, there were a total of 703 instances of acute reactions, out of which 44 cases were classified as anaphylaxis. PEG or PS80 were pre-sent as excipients in 353 of these cases, accounting for 28.8% of the content.

An antibiotic was involved in 468 occurrences, followed by non-steroidal anti-inflammatory drugs in 342 cases, laxatives in 16 cases, chemotherapy agents in 21 cases, and contrast agents in 251 cases.

A total of 378 patients were diagnosed with asthma, with approximately half of them (192 pa-tients, or 50.7%) having a non-controlled state. However, only 108 patients (28.5%) received background therapy. Four patients were found to have severe asthma.

At the time of the visit, 44 out of 67 cases of chronic spontaneous urticaria were under control, whilst a history of episodic urticaria was detected in 120.

One hundred-three high-risk patients were referred by the Allergy Call Center, while 112 of them (13%) were sent by other physicians (X 54, *p* < 0.00001). Among the former group, one had mastocytosis, eight had uncontrolled chronic spontaneous urticaria, and nineteen had un-controlled asthma. In 42 patients (41%) and 58 patients (56%), respectively, the visit was re-quested as an adverse reaction to a drug containing PEG or to the anti-COVID-19 vaccine was reported.

At the time of the initial visit, 308 patients (25.2%) were already administered at least one dose of vaccination, with a report of reactions in 294 cases. Amongst them, 238 patients (77.2%) re-ceived Comirnaty/Biontech, 45 patients were vaccinated with Moderna (14.6%), and 24 (7.7%) of them were administered VaxZevria/AstraZeneca. Jcovden/Janssen was used in only 1 patient (0.3%).

More than half of the reactions were immediate: 140 reactions (47.6%) occurred within an hour of vaccination, 38 (12.9%) between 1 and 6 h; 116 cases were of non-immediate reactions (39.4%), 53 of which occurred between 6 and 12 h, and 63 reactions occurred after more than 12 h. The most common immediate reactions were urticaria (49 patients, 16.6%), pruritus (38 patients, 12.9%), and dyspnea (42 patients, 14.2%). Only one case of reaction con-sistent with anaphylaxis was reported. In 57% of the cases, the reaction regressed within 24 h.

## Discussion

Communication between health authorities and healthcare providers is essential for public health emergencies. Telephone or online support can simplify such communications, but little data are available on the methods used by health authorities to support healthcare workers (HCWs) during the COVID-19 pandemic, including data regarding the activity of specific call cen-tres.

The World Health Organization included online portals as a mean of communication with HCWs. [17] and has updated its information platform with the registry about allergic reactions to anti-COVID vaccines [18].

The US Centers for Disease Control and Prevention provided a comprehensive website contain-ing various resources for healthcare providers. Additionally, they conducted regular 'Clinical Outreach and Communication' calls and webinars to offer training for HCWs. Furthermore, they activated a 'Clinician On-Call Center' that can be accessed by telephone for healthcare providers [19].

The National Health Service (NHS) in England provided a helpline for healthcare workers, which can be accessed via telephone or text message [20]. No published reports are available to describe the effects of different communication methods adopted by the authorities to support healthcare providers in the community. Call centres have already demonstrated their ability to offer a proper level of support in many fields, i.e. reducing the number of surgery contacts and overtime visits by general practitioners [21]. Telephone consultations can also provide the opportunity to discuss the new treatment approaches as they can facilitate both the development and the application of new guidelines [15].

The implementation of the ACC in the anti-SARS-CoV-2 vaccination campaign in our area can be regarded as an exemplary approach as it could prove its helpfulness in crisis (namely pandemics or situations which require to process a considerable amount of clinical data in a short timeframe); in fact, among the over 15,000 telephone inquiries that were attended, only 336 vis-its were performed by allergists.

The need for patient stratification led to a significant request for allergy visits; the percentage of patients who were never visited by an allergist in our cohort is rather comparable to those found in other countries [22] as they were both approximately 60%. About 80% of the subjects were referred because they reported a previous reaction to drugs, particularly antibiotics and anti-inflammatories; however, less than a third of the related drugs actually contained either PEG or PS80.

In the asthmatic population of our study, disease control occurred in only half of the subjects; however, adherence to background therapy merely occurred in a third of our population [23]; this element appears to be coherent with the cohorts found in the literature.

The ACC assessments proved to be effective in rapidly selecting high-risk patients among wide populations; its cost-effectiveness ratio and the screening procedures were demonstrated in clinical practice as it helped all the healthcare providers to better and faster support patients and co-workers.

This is the very first example of a large-scale mechanism which tried to help both clinicians and the national health system to avoid an overload given by a high number of unnecessary re-quests. This approach could be further emulated and expanded as to filter and better assess pa-tients not only in emergencies (ie: the COVID-19 pandemic) but throughout all the clinical path-ways established by the national health systems.

## Conclusions

A pre-emptive structure for the screening of patients at risk for the development of allergic re-actions, like the one offered by our Allergy Call Center, appeared to be effective in the evaluation and management of a high volume of requests without sacrificing the quality or the quantity of the consultations.

This approach led to a prominent decrease in allergological visits to our tertiary care Centre, reducing the waiting times and providing additional support for both patients and healthcare providers, thus allowing the vaccinations to be more easily handled.

## Supplementary Information

Below is the link to the electronic supplementary material.

Supplementary file1 (DOCX 22 kb)

Supplementary file2 (PDF 1248 kb)

## Data Availability

No datasets were generated or analysed during the current study.
