# Supplementary material for: How a Novel Approach of Allergy Call Center Improved the Management of the Anti-COVID Vaccination Campaign in Piedmont: Italy
Source: J Epidemiol Glob Health. 2024 Oct 14;14(4):1764–70. doi: 10.1007/s44197-024-00309-2 (PMC11652545; doi:10.1007/s44197-024-00309-2)
Supplement: Supplementary file 1 — Supplementary file1 (DOCX 22 kb) [file 44197_2024_309_MOESM1_ESM.docx]

Procedure Diagram: Patient referral and risk stratification procedure for vaccination management


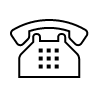

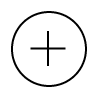

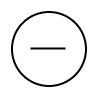


General Practitioners (499 patients)

Internal Medicine consultants (95 patients)

Occupational medicine physicians (61 patients)

Other medical experts (231 patients)

Vaccinating Physicians

Allergy Call Centre

15865 calls

Immunology and Allergy Unit,

Azienda Ospedaliera

Ordine Mauriziano, Turin

(1222 patients)

886 patients

336 patients

Risk stratification

Low risk

Moderate-high risk

Vaccination without limitations

Therapy optimisation

Allergy test for excipients

Vaccination with an alternative vaccine or complete exemption

Vaccination with an eventual indication of observation time and/or specific setting
