# Supplementary material for: How a Novel Approach of Allergy Call Center Improved the Management of the Anti-COVID Vaccination Campaign in Piedmont: Italy
Source: J Epidemiol Glob Health. 2024 Oct 14;14(4):1764–70. doi: 10.1007/s44197-024-00309-2 (PMC11652545; doi:10.1007/s44197-024-00309-2)
Supplement: Supplementary file 2 — Supplementary file2 (PDF 1248 kb) [file 44197_2024_309_MOESM2_ESM.pdf]

Azienda Ospedaliera
Ordine Mauriziano
di Torino

Sede legale: Via Magellano, 1 - 10128 TORINO - Tel. +39 011.508.1111 - www.mauriziano.it - P.I./Cod. Fisc. 09059340019

DIPARTIMENTO AREA MEDICA
S.S.D. D.U. – IMMUNOLOGIA CLINICA ed ALLERGOLOGIA
Dirigente Responsabile: Prof. Giovanni ROLLA
Largo Turati, 62 Torino
Telefono: 011/508. 2421 / 2926
e-mail: grolla@mauriziano.it

PROTOCOLLO OPERATIVO PER IL
RISCHIO ALLERGOLOGICO ALLA SOMMINISTRAZIONE DEI VACCINI
PER SARS-COV2 ATTUALMENTE IN COMMERCIO

L'incidenza di reazioni anafilattiche al vaccino per SARS-Cov2 Pfizer-BioNTech si attesta attualmente su 5/1000000, ai vaccini Moderna e AZ, attualmente è di circa 3/1000000, di poco superiore alla media degli altri vaccini antimicrobici. Questi valori sono probabilmente destinati a modificarsi con il procedere della campagna vaccinale. (*Nature News 16/2/2021*)

AIFA ha ricevuto 14 segnalazioni di anafilassi al Vaccino Pfizer (Comirnaty), su 1564000 dosi, con un'incidenza stimata di 8 casi per milione (AIFA - *rapporto 1 sulla sorveglianza dei vaccini COVID-19, periodo 27/12/2020-26/01/20201*). La patogenesi di queste reazioni non è ancora chiarita; i sospetti maggiori riguardano il polietilenglicole (PEG) 2000, una molecola polimerica utilizzata come stabilizzatore del layer idrofilico, eccipiente presente nei 2 vaccini mRNA, il polisorbato 80 presente nel vaccino AZ ed è stato recentemente identificato anche il trometamolo, eccipiente presente nel vaccino ad mRNA Moderna.

Il fattore di rischio più importante finora identificato per le reazioni gravi da ipersensibilità è rappresentato da pregresse reazioni da ipersensibilità a vaccini e farmaci contenenti PEG o Polisorbato e medicamenti o mezzi di contrasto contenenti trometamolo.

La task-force statunitense (*JACI in Practice 2021*) ha elaborato un questionario (5 domande) per la stratificazione del rischio di reazioni gravi di ipersensibilità al vaccino per SARS-COV2. Tale questionario è stato ripreso e in parte modificato dalle società italiane di allergologia e immunologia clinica (AAIITO, SIAAIC).

Questo documento ha i seguenti obiettivi:

1. Fornire un algoritmo di semplice interpretazione per selezionare i pazienti ad alto, medio e basso rischio di anafilassi ai due vaccini COVID-19 attualmente in commercio, con in appendice un elenco di medicinali contenenti gli eccipienti suddetti, in modo da effettuare un primo screening, e di selezionare i pazienti meritevoli di un consulto da parte di uno specialista allergologo, propedeutico alla somministrazione del vaccino stesso.
2. Valutare l'indicazione alla somministrazione della seconda dose di vaccino in pazienti che hanno sviluppato segni e/o sintomi compatibili con reazione allergica alla prima dose dello stesso.

Fonte: *Mass General Brigham and Vanderbilt allergy expert consensus prior to initial COVID-19 vaccination – modificato*; *Nature News 16/2/2021; AIFA rapporto 1 sulla sorveglianza dei vaccini COVID-19, periodo 27/12/2020-26/01/20201*); *JACI in Practice 2021; Linee guida* AAIITO, SIAAIC

RACCOLTA ANAMNESTICA CONSIGLIATA

PER VALUTAZIONE DEL PAZIENTE CANDIDATO ALLA VACCINAZIONE PER SARS-CoV-2

NON DEVONO essere considerate motivo di richiesta di CONSULTO ALLERGOLOGICO le seguenti condizioni:

- **Rinite allergica**
- **Asma bronchiale lieve/moderata controllata**
- **Allergia alimentare**
- **Orticaria cronica spontanea**

Sono motivo di richiesta di consulenza allergologica le Reazioni gravi da ipersensibilità, specie verso farmaci/vaccini contenenti gli eccipienti PEG o polisorbato e medicamenti o mezzi di contrasto contenenti trometamolo

In generale,

Il Medico di Medicina Generale/Vaccinatore deve verificare le seguenti condizioni, al fine di valutare la reale indicazione a visita allergologica:

- **Pregresse reazioni allergiche *gravi* idiopatiche** (es. orticaria diffusa/angioedema in associazione a sintomi sistemici, dispnea, broncospasmo costrizione al giugulo, addominalgie/diarrea, ipotensione), insorte senza apparente agente scatenante in cui sia stata diagnosticata l'Anafilassi Idiopatica da parte dello Specialista Allergologo. Somministrazione della vaccinazione per SARSCoV-2 **in ambiente ospedaliero**; raccomandata osservazione 60' dopo la somministrazione.

Nei casi dubbi (paziente non ancora valutato dallo Specialista Allergologo) **è raccomandata consulenza allergologica** prima della vaccinazione.

- **Pregresse reazioni allergiche *gravi* a farmaci**: il MMG/vaccinatore valuterà principio attivo ed eccipienti della molecola ritenuta responsabile della reazione avversa ed effettuerà attenta analisi della terapia domiciliare del paziente*. Se tra gli eccipienti delle molecole che hanno causato la reazione grave sono contenuti/segnalati PEG (macrogol) oppure polisorbato **è raccomandata consulenza allergologica**. Se tra le molecole tollerate dal paziente sono segnalati PEG (macrogol) oppure polisorbato, non è controindicata la vaccinazione anti SARS- CoV-2.

- **Pregresse reazioni allergiche *gravi* ad altre vaccinazioni**: il MMG valuterà principio attivo ed eccipienti della vaccinazione ritenuta responsabile della reazione avversa ed effettuerà attenta analisi della terapia domiciliare del paziente*. Se tra gli eccipienti delle molecole che hanno causato la reazione grave sono contenuti/segnalati PEG (macrogol) oppure polisorbato **è raccomandata consulenza allergologica**. Se tra gli eccipienti delle molecole tollerate dal paziente sono segnalati PEG (macrogol) oppure polisorbato, non è controindicata la vaccinazione anti SARS- CoV-2.

- **Pregresse reazioni allergiche *gravi* a**:

  - ***Alimenti*: non è indicata visita allergologica**. Somministrazione della vaccinazione per SARS-CoV-2 in ambiente standard; raccomandata osservazione 60' dopo la somministrazione.

- ***Lattice:*** **non è indicata visita allergologica**. Somministrazione della vaccinazione per SARS-CoV-2 in ambiente latex-safe e con DPI latex-free; raccomandata osservazione 60' dopo la somministrazione.

- ***Imenotteri:*** **se il paziente non è affetto da mastocitosi** **, **non è indicata visita allergologica**. Somministrazione della vaccinazione per SARS-CoV-2 in ambiente standard; raccomandata osservazione 60' dopo la somministrazione.

- **Pregresse reazioni allergiche *gravi* a sostanze contenenti PEG, polisorbato, polyoxyl 35 castor oil o trometamolo: è raccomandata consulenza allergologica.**

CASI PARTICOLARI

- Asma non controllata: *** Se il paziente presenta un'asma non controllata, è necessario rimandare la vaccinazione e impostare adeguato trattamento per la patologia di base.

- Asma non controllata nonostante ottimizzazione della terapia: è necessario eseguire la vaccinazione **in ambiente ospedaliero** con osservazione di 60'.

- Asma controllata: non è indicata visita allergologica. Somministrazione della vaccinazione per SARS-CoV-2 in ambiente standard; raccomandata osservazione 15' dopo la somministrazione.

- Orticaria cronica spontanea: non indicata visita allergologica. È raccomandato un adeguato trattamento per il controllo dell'orticaria spontanea. Somministrazione della vaccinazione per SARS-CoV-2 in ambiente standard; raccomandata osservazione 15' dopo la somministrazione.

* Attenta analisi della terapia domiciliare del paziente:

Farmaci recentemente assunti contenenti PEG o macrogol tra gli eccipienti: si vedano tabelle 2, 3, 4 (NB: *elenco incompleto. Si raccomanda di eseguire accurata ricerca degli eccipienti di ogni singolo farmaco assunto dal paziente*).

Farmaci recentemente assunti contenenti polisorbato tra gli eccipienti: si vedano tabelle 2, 3, 4 (NB: *elenco incompleto. Si raccomanda di eseguire accurata ricerca degli eccipienti di ogni singolo farmaco assunto dal paziente*).

*****Mastocitosi*** o sindrome da attivazione mastocitaria: se il paziente è affetto da mastocitosi, è necessario effettuare premedicazione con cetirizina 10 mg gocce o compresse (XX gocce o 1 co) da assumere il giorno precedente, il giorno della vaccinazione (entro le 10 ore prima dell'ora prevista per la somministrazione) e 1 co o XX gtt/die i 4 giorni a seguire. Il paziente deve portare con sé la penna di adrenalina autoiniettabile. Somministrazione della vaccinazione per SARS-CoV-2 in ambiente ospedaliero; raccomandata osservazione 60' dopo la somministrazione.

Se il paziente ha una diagnosi di mastocitosi e ha manifestato una pregressa reazione da ipersensibilità immediata (anafilassi o orticaria) temporalmente correlabile ad altre vaccinazioni oppure una anafilassi idiopatica (reazione allergica grave senza apparente agente scatenante), è raccomandato prolungare l'osservazione post-vaccinale ad almeno quattro ore e la

somministrazione della vaccinazione deve essere eseguita in presenza di un medico rianimatore (Cfr. Fig. 3).

***_**Asma non controllata**_ (presenza di uno o più dei seguenti criteri):

Nelle ultime 4 settimane il paziente ha avuto:

- Sintomi diurni di asma più di 2 volte a settimana
- Risvegli notturni dovuti all'asma
- Necessità di utilizzare il farmaco d'emergenza (broncodilatatore) più di 2 volte a settimana
- Ha avuto qualche limitazione delle proprie attività quotidiane dovute all'asma

Interpretazione:

- Asma ben controllata: nessuno dei suddetti sintomi
- Asma parzialmente controllato: 1-2 dei suddetti sintomi
- Asma non controllato: 3-4 dei suddetti sintomi

Fig.1 Stratificazione del rischio di reazioni allergiche alla prima somministrazione di vaccino SARS-Cov-2

ANAMNESI DEL PAZIENTE

1. Storia di reazioni allergiche gravi/anafilassi (Fig. 4) ad un **farmaco** (es. orticaria diffusa/angioedema in associazione a sintomi sistemici (dispnea, broncospasmo costrizione al giugulo, addominalgie/diarrea, ipotensione)
2. Storia di reazioni allergiche gravi/anafilassi ad un **vaccino.**
3. Storia di reazioni allergiche gravi/anafilassi ad **alimenti, veleno di imenotteri o lattice.**
4. Storia di reazioni allergiche gravi/anafilassi a **Polietilenglicole (PEG), polisorbato, polyoxyl 35 castor oil** (es. Paclitaxel) o **trometamolo[§]**.
5. Paziente affetto da **mastocitosi o sindrome da attivazione mastocitaria** (**vedere Fig. 3**)

RACCOMANDAZIONI PER LA PRIMA DOSE DI VACCINO

Presenza criterio n°4

→ **VACCINAZIONE CONTROINDICATA**

→ **VALUTAZIONE ALLERGOLOGICA**

- Anafilassi verso un farmaco o vaccino contenente PEG, polisorbato o polyoxyl 35 castor oil (Tab. 2-3-4)

→ **RISCHIO ELEVATO**

→ **VALUTAZIONE ALLERGOLOGICA**

- Asma bronchiale non controllato nonostante ottimizzazione della terapia;
- Anafilassi idiopatica (senza causa nota);

→ **RISCHIO INTERMEDIO**

→ **VACCINAZIONE CON 60 MIN. DI OSSERVAZIONE IN AMBIENTE OSPEDALIERO**

- Anafilassi verso cibi, veleno di imenotteri o lattice;
- Anafilassi a farmaci o vaccini non contenenti PEG, polisorbato o polyoxyl 35 castor oil;

→ **RISCHIO INTERMEDIO**

→ **VACCINAZIONE CON 60 MIN. DI OSSERVAZIONE**

Tutti i criteri negativi:

- Reazioni allergiche non anafilattiche a farmaci/vaccini, alimenti, veleno di imenotteri o lattice;
- Rinite allergica e/o asma bronchiale controllato.
- Orticaria cronica spontanea

→ **RISCHIO BASSO**

→ **VACCINAZIONE CON 15 MIN. DI OSSERVAZIONE**

[§]**trometamolo**: se pregresse reazioni gravi/anafilassi a mezzo di contrasto per RM a base di gadolinio e/o mezzi di contrasto iodato e/o farmaci/medicamenti contenenti trometamolo

Fig.2: Stratificazione del rischio dopo reazione allergica alla prima dose di vaccino SARS-Cov-2.

REAZIONE ALLERGICA DOPO LA PRIMA DOSE DI VACCINO

Anamnesi, parametri vitali, esame obiettivo, imaging, es. di laboratorio*, trattamento.

RISCHIO ELEVATO

- Anafilassi dopo la prima dose del vaccino

RISCHIO INTERMEDIO

- Coinvolgimento muco-cutaneo (orticaria acuta, angioedema, prurito diffuso)
- Sintomi/segni respiratori isolati (dispnea, tosse, sibili/broncospasmo, oppressione toracica, desaturazione)

RISCHIO BASSO

- Reazione locale al sito di iniezione;
- Segni e sintomi non compatibili con patologia allergica.

Seconda dose con 30 min di osservazione

<u>VALUTAZIONE SPECIALISTICA ALLERGOLOGICA CON EVENTUALE ESECUZIONE DI TEST CUTANEI</u>

*In particolare dosaggio della triptasi sierica entro 2 ore dalla reazione.

FIG 3. Criteri per la gestione del paziente con mastocitosi

**Tutti** i pazienti con diagnosi di mastocitosi				
**Premedicazione**	Farmaco	Dose	Quando	Vaccinazione
	Cetirizina gocce o compresse	XX gocce o 1 co	- il giorno precedente<br>- il giorno della vaccinazione (entro le 10 ore prima dell'ora prevista per la somministrazione)<br>- 1 co o XX gtt/die i 4 giorni a seguire	In ambiente ospedaliero.<br>Osservazione di **60'**.<br>Penna di **adrenalina** con sé
Paziente con **mastocitosi E pregressa anafilassi** o orticaria temporalmente correlabile ad altre **vaccinazioni** oppure una anafilassi **idiopatica**				
**Premedicazione**	Farmaco	Dose	Quando	Vaccinazione
	Cetirizina gocce o compresse	XX gocce o 1 co	- il giorno precedente<br>- il giorno della vaccinazione (entro le 10 ore prima dell'ora prevista per la somministrazione)<br>- 1 co o XX gtt/die i 4 giorni a seguire	In ambiente ospedaliero.<br>Osservazione di almeno 4 **ore**.<br>Penna di **adrenalina** con sé.<br>Presenza del **medico rianimatore** durante vaccinazione.

FIG 4. Criteri per la diagnosi di anafilassi (reazione allergica grave)

L'anafilassi è probabile quando viene soddisfatto uno dei 3 seguenti criteri:

1

Insorgenza acuta dei sintomi (da minuti a qualche ora) con coinvolgimento di:

- **Cute e/o mucose**
  - Prurito
  - Flushing
  - Pomfi
  - Angioedema

ASSOCIATO A

- **Sintomi respiratori**
  - Dispnea
  - Sibili/broncospasmo
  - Riduzione del PEF
  - Stridore
  - Ipossiemia

OPPURE

- **Riduzione PAO o disfunzione d'organo**
  - Sincope
  - Incontinenza
  - Collasso cardiocircolatorio

2

Insorgenza rapida di **due o più dei seguenti sintomi** dopo esposizione ad un allergene probabile:

- **Cute e/o mucose**
  - Prurito
  - Flushing
  - Pomfi
  - Angioedema
- **Sintomi respiratori**
  - Dispnea
  - Sibili/broncospasmo
  - Riduzione del PEF
  - Stridore
  - Ipossiemia
- **Riduzione PAO o disfunzione d'organo**
  - Sincope
  - Incontinenza
  - Collasso cardiocircolatorio
- **Sintomi gastrointestinali persistenti:**
  - Vomito
  - Crampi addominali
  - Diarrea

3

Dopo **l'esposizione ad un allergene noto** per il paziente (da pochi minuti a qualche ora):

- **Ipotensione**

Diagnosi differenziale tra anafilassi, reazione vasovagale e sindrome da iperventilazione.

**Caratteristiche cliniche**	**Anafilassi**	**Reazione Vasovagale**	**Sindrome da iperventilazione**
Latenza di insorgenza dopo l'iniezione	Entro 30 minuti dalla somministrazione (reazioni gravi solitamente entro 15 minuti)	Solitamente pochi secondi fino a qualche minuto dopo la somministrazione (raramente prima)	Latenza variabile, con progressione rapida
Polso	Rapido, debole, irregolare	Lento, debole ma regolare	Rapido, ampio, regolare
Pressione arteriosa	Ipotensione (pressione sistolica <90 mmHg), possibile progressione verso collasso cardiocircolatorio	Ipotensione transitoria	Pressione normale o lieve ipertensione
Coinvolgimento neurologico	Da malessere generalizzato a perdita di coscienza	Lipotimia, vertigine, talora perdita di coscienza	Parestesie (formicolio periorale), agitazione, vertigini, cefalea, tetania periferica
Coinvolgimento respiratorio	Dispnea, tosse, sibilo toracico, stridore	Frequenza respiratoria ridotta, talora apnea di breve durata	Tachipnea, iperpnea, dispnea, laringospasmo (senso di costrizione al giugulo)
Coinvolgimento cutaneo	Prurito diffuso, orticaria, angioedema;	Diaforesi, cute pallida	Assente
Coinvolgimento gastrointestinale	Nausea, vomito, dolori addominali, diarrea	Nausea, vomito	Assente
Terapia	Vedere raccomandazioni WAO sottostanti	Posizionare il paziente in Trendelenburg. Areare la stanza. Apporre un panno freddo e umido sulla fronte. Rassicurare il paziente	Rassicurare il paziente. Chiedere al paziente di respirare in modo lento e regolare

Strumenti e farmaci necessari per la gestione di una anafilassi

EQUIPAGGIAMENTO NECESSARIO IN OGNI SITO VACCINALE	EQUIPAGGIAMENTO PER CENTRI VACCINALI OSPEDALIERI
**Adrenalina in siringa pre-riempita 300 mcg 1 fl (Chenpen)** e adrenalina soluzione 1:1000 (1 mg/ml), 2 fiale	Defibrillatore + Piastre del defibrillatore
**Antistaminici:** clorfenamina (Trimeton 10 mg/ml): 3 fl	Laringoscopio e componenti di ricambio
**Corticosteroidi**: metilprednisolone (Urbason 40 mg/ml): 3 flaconi	Materiale per l'intubazione
**Soluzione fisiologica**: 5 flac da 500 ml	Ambu
**Sfigmomanometro e Fonendoscopio**	Maschera tascabile per adulto con valvola unidirezionale (maschera per rianimazione cardiopolmonare (RCP))
Broncodilatatore (es. Salbutamolo)	
Guanti sterili e non sterili	
Bombola d'ossigeno e maschera	
Materiale per accesso venoso periferico	
Siringhe con aghi da intramuscolo 20-22 G	

LA TERAPIA ANTIISTAMINICA E' DA CONSIDERARSI IN AGGIUNTA ALL'ADRENALINA, MA NON DEVE ESSERE SOMMINISTRATA COME PRIMO O COME UNICO FARMACO

PRESTARE PARTICOLARE ATTENZIONE NEL SOMMINISTRARE FARMACI PER OS A PAZIENTI CON OSTRUZIONE DELLE VIE AEREE INGRAVESCENTE

Raccomandazione World Allergy Organization (WAO) per la gestione dell’anafilassi

**Valutazione generale**	
**I** – Avere un adeguato protocollo scritto per il riconoscimento e trattamento dell’anafilassi (cfr fig.4)	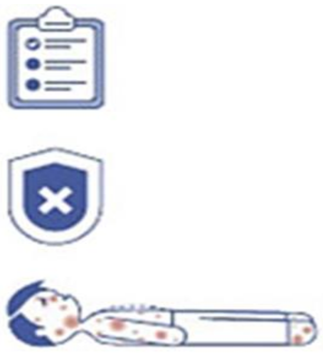
**II** – Rimuovere i possibili fattori scatenanti (es. farmaci in corso che possano avere causato la reazione)	
**III** – Valutazione del paziente:<br>A – Airways / B – Breathing / C – Circulation<br>Stato di coscienza, cute, peso corporeo	

**GESTIONE DELL’ANAFILASSI**	
**1 - Paziente in posizione supina e con gambe sollevate**	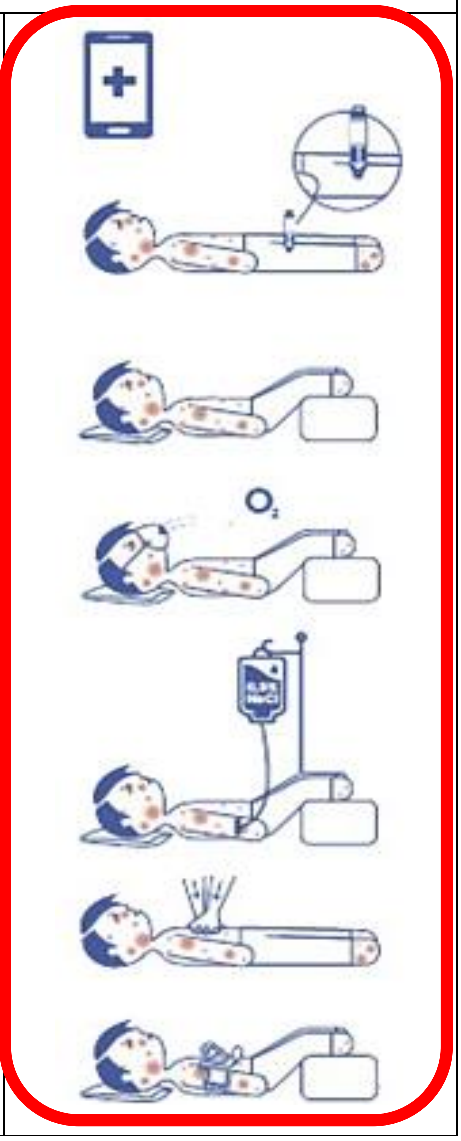
**2 – Somministrazione di ADRENLINA** (muscolo vasto laterale della coscia): 0.01 mg/kg di soluzione 1:1000 (1 mg/ml) **0.5 ml adulti – 0.3 ml bambini. Ripetere dopo 5-15 minuti.**	
**2a – contestualmente reperire accesso venoso (14-16G). Somministrare NaCl 0.9% 1-2 litri (5-10 mg/kg nei primi 5-10’)**	
**3 – Se indicato, O2 terapia con maschera facciale 6-8 litri/min**	
**4 – Chiamare i soccorsi - 112**	
**5- Se necessario, effettuare rianimazione cardio-polmonare**	
**6– monitoraggio costante dei parametri vitali (PAO, FC, FR, SatO2)**	
**7- Se ipotensione/shock persistente:**<br>- **soluzione fisiologica** (massimo 50 ml / kg nei primi 30 minuti)<br>- Bolo di **glucagone** per via endovenosa di 1-2 mg negli adulti, 20-30 microgrammi / kg fino a 1 mg nei bambini. Questo può essere ripetuto o seguito da un'infusione di 1-2 mg / ora negli adulti.	

Misure aggiuntive da considerare se l'infusione di adrenalina IM è inefficace

ASCIA 2020 Acute management of anaphylaxis guidelines

Per l'ostruzione delle vie aeree superiori:

Adrenalina nebulizzata (5 ml ad es. 5 fiale da 1:1000).

Per broncospasmo persistente:

Salbutamolo 8-12 erogazioni da 100 µg (spaziatore) o 5 mg (nebulizzatore).

Antistaminici e corticosteroidi

Antistaminici:

Nessuna utilità nel trattamento dell'anafilassi, ma utili per alleviare alcuni sintomi.

Clorfenamina 10 mg/ml: 1 fl im.

Corticosteroidi:

Il beneficio dei corticosteroidi nell'anafilassi non è dimostrato.

Metilprednisolone 40 mg/ml: 1 fl ev.

Tabella 1. Componenti dei vaccini anti SARS-Cov-2 (Pfizer-BioNTech/Moderna/AstraZeneca)

	**Pfizer-BioNTech**	**Moderna**
Attivi	RNA messaggero con nucleosidi modificati (modRNA) codificante la glicoproteina virale "spike" (S) del virus SARS-CoV2	mRNA con nucleosidi modificati codificante la glicoproteina virale "spike" (S) del virus SARS-CoV2
Inattivi - lipidi	(4-idrossibutil)azandiil)bis(esano-6,1-diil) bis (2-esildecanoato)	SM-102 (Di proprietà di Moderna)
	2[(Polietilenglicole [PEG])-2000]-N, N-ditetradecilacetammide	Polietilenglicole (PEG) 2000 dimiristoil glicerolo (DMG)
	1,2-distearoil-sn-glicero-3-fosfocolina	1,2-distearoil-sn-glicero-3-fosfocolina
	Colesterolo	Colesterolo
Inattivi – Sali, Disaccaridi, Soluzioni tampone	Cloruro di potassio, fosfato di potassio monobasico, cloruro di sodio, sodio fosfato bibasico diidrato	Trometamina, trometamina idrocloride, acido acetico, sodio acetato
	Disaccaride (saccarosio)	Disaccaride (saccarosio)
	Diluente (Cloruro di sodio)	Diluente (Nessuno)

Tabella 2. Elenco esemplificativo degli eccipienti polisorbato e polietilenglicole contenuti in vaccini.

**Eccipienti**	**Tipologia Vaccino**	**Vaccino**	**Quantità eccipiente per dose**
Polisorbato 20	Influenza	Flublok&Flublock quad	<27.5 mcg (Tween20)
	Epatite A	Havrix	0.05 mg/ml
	Epatite A&B	Twinrix	Sconosciuta
	Sars-CoV-2 (Sanofi)		
Polisorbato 80	Tdap	Boostrix	<100 mcg (Tween 80)
	Influenza	Fluad	1.175 mg
	Influenza	Fluarix quad	<0.0550 mg (Tween 80)
	Influenza	Flucelvax quad	<1500 mcg (Tween 80)
	Influenza	Flulaval Quad	<887 mcg
	HPV	Gardasil and Gardasil-9	50 mcg
	Epatite B	Heplisav-B	
	DTaP	Infanrix	0.1 mg/ml
	Encefalite Giapponese	JE-Vax	<100 mcg (Tween 80)
	DTaP + IPV	Kinrix	<0.0007%
	DTaP+HepB+IPV	Pediarix	<100 mcg (Tween 80)
	Anti-Pneumococcico 13 valente	Prevnar 13	<100 mcg (Tween 80)
	DTaP + IPV	Quadracel	100 mcg
	Rotavirus	RotaTeq	10 ppm
	Zoster	Shingrix	?
	Meningococco gruppo B	Trumenba	0.08 mg
	DTaP+IPV+HepB+Hib	Vaxelis	0.018 mg
	Sars-CoV-2 (Astrazeneca)		<0.0056%
	Sars-CoV-2 (Johnson&Johnson)		
PEG2000	Sars-CoV-2 (Moderna)		
	Sars-CoV-2 (Pfizer)		

Tabella 3. Elenco esemplificativo (incompleto) di farmaci iniettabili contenenti polietilenglicole (PEG)

Nome generico (**Nome commerciale**)	Peso Molecolare	Descrizione generica
Metilprednisolone acetato (**Depo-Medrol)**	PEG 3350	Glucocorticoide antinfiammatorio per iniezioni intramuscolo, intra-articolari, in tessuti molli o intralesionale.
Metossipolietilenglicole-epoetina beta (**Micera**)	30 kD Metossi polietilene acido butanoico glicolico	Trattamento dell'anemia negli adulti con malattia renale cronica (CKD).
Pegfilgrastim (**Neulasta**)	20 kD Monometossi polietilenglicole	Utilizzato per ridurre la possibilità di infezione data da una bassa conta di globuli bianchi, in persone con determinati tipi di cancro (non mieloide), che ricevono farmaci antitumorali (chemioterapia) che possono causare febbre e una bassa conta ematica di globuli bianchi.
Medrossiprogesterone acetato (**Depo-Provera**)	PEG 3350	Terapia contraccettiva e adiuvante e trattamento palliativo di carcinomi renale ed endometriale inoperabili,ricorrenti e metastatici.
Brilliant Blue G Soluzione oftalminca (**TissueBlue**)	PEG 3350	Colorante selettivo della membrana limitante interna (ILM).
Esafluoruro di zolfo (**Lumason**)	PEG 4000	Mezzo di contrasto per ecografie.
Impianto bimatoprost (**Durysta**)	PEG non specificato	Riduzione della pressione intraoculare (IOP) nei pazienti con glaucoma ad angolo aperto (OAG) o ipertensione oculare (OHT).

Trastuzumab (**Herceptin, Herzuma, Kanjinti, Ogivri, Ontruzant**)	PEG 3350	Trattamento adiuvante del carcinoma della mammella con overespressione di HER2 e con presenza o meno di metastasi linfonodali.
Rilonacept (**Arcalyst**)	PEG 3350	Inibitore dell'interleuchina-1 per il trattamento delle Sindromi periodiche associate alla criopirina (CAPS).
Microsfera lipidica Perflutren (**Definity**)	PEG 5000	Mezzo di contrasto utilizzato durante l'ecocardiogramma per schiarire e definire le immagini del cuore.

Tabella 4. Elenco esemplificativo (incompleto) di farmaci iniettabili contenenti polisorbato.

Classe di Farmaci	Nome generico (**Nome commerciale**)	Polisorbato
Antiaritmici	Amiodarone idrocloride (solo generici)	Polisorbato 80
Ipoglicemizzanti	Exenatide (**Bydureon Bcise**)<br>Insulina glargine (**Lantus, Semglee**)<br>Insulina glulisina (**Apidra**)<br>Dulaglutide (**Trulicity**)	Polisorbato 20<br>Polisorbato 20<br>Polisorbato 20<br>Polisorbato 80
Antidoti	Ialuronidasi (**Hylenex Recombinant)**	Polisorbato 80
Antifungini	Anidulafungina (**Eraxis**)	Polisorbato 80
Antineoplastici	Ofatumumab (**Kesimpta**)<br>Siltuximab (**Sylvant**)	Polisorbato 80<br>Polisorbato 80
Antipsicotici	Palmitato paliperidone (**Invega Trinza, Invega Sustenna**)<br>Lauroxil aripiprazolo (**Aristada**)	Polisorbato 20<br>Polisorbato 20
Antiretrovirali	Ibalizumab (**Trogarzo**)	Polisorbato 80
Antipsoriasici	Adalimumab (**Humira, Imraldi**)<br>Golimumab (**Simponi**)<br>Guselkumab (**Tremfya**)<br>Infliximab - dyyb (**Inflectra, Remicade, Renflexis**)<br>Ustekinumab (**Stelara**)	Polisorbato 20 (Imraldi) - Polisorbato 80 (Humira)<br>Polisorbato 80<br>Polisorbato 80<br>Polisorbato 80<br>Polisorbato 80
Antivirali	Interferon alfa-2b (**Intron A**)	Polisorbato 80
Modificanti la risposta biologica	Interferon gamma-1b (**Actimmune**)	Polisorbato 20
Trattamenti oncologici	Ado-trastuzumab emtansine (**Kadcyla**)<br>Atezolizumab (**Tecentriq**)<br>Avelumab (**Bavencio**)<br>Bevacizumab (**Avastin, Zirabev**)<br>Daratumumab/hyaluronidase (**Darzalex Faspro**)	Polisorbato 20<br>Polisorbato 20<br>Polisorbato 20<br>Polisorbato 20<br>Polisorbato 20

	Denosumab (**Prolia, Xgeva**)	Polisorbato 20
	Dinutuximab (**Unituxin**)	Polisorbato 20
	Enfortumab (**Padcev**)	Polisorbato 20
	Olaratumab (**Lartruvo**)	Polisorbato 20
	Palifermin (**Kepivance**)	Polisorbato 20
	Pertuzumab/trastuzumab/hyaluronidase (**Phesgo**)	Polisorbato 20
	Polatuzumab vedotin (**Polivy**)	Polisorbato 20
	Tafasitamab (**Monjuvi**)	Polisorbato 20
	Trastuzumab (**Herceptin, Herceptin Hylecta, Herzuma, Kanjinti, Ontruzant, Trazimera**)	Polisorbato 20
	Belantamab (**Blenrep**)	Polisorbato 80
	Brentuximab vedotin (**Adcetris**)	Polisorbato 80
	Cemiplimab (**Libtayo**)	Polisorbato 80
	Docetaxel (**Taxotere**)	Polisorbato 80
	Durvalumab (**Imfinzi**)	Polisorbato 80
	Elotuzumab (**Empliciti**)	Polisorbato 80
	Etoposide (**Toposar, VePesid**)	Polisorbato 80
	Fam-trastuzumab deruxtecan (**Enhertu**)	Polisorbato 80
	Fosaprepitant dimeglumine (**EMEND, Fosaprepitant**)	Polisorbato 80
	Inotuzumab ozogamicin (**Besponsa**)	Polisorbato 80
	Ipilimumab (**Yervoy**)	Polisorbato 80
	Isatuximab (**Sarclisa**)	Polisorbato 80
	Mogamulizumab (**Poteligeo**)	Polisorbato 80
	Moxetumomab pasudotox (**Lumoxiti**)	Polisorbato 80
	Nivolumab (**Opdivo**)	Polisorbato 80
	Ofatumumab (**Arzerra**)	Polisorbato 80
	Pembrolizumab (**Keytruda**)	Polisorbato 80
	Ramucirumab (**Cyramza**)	Polisorbato 80
	Rituximab (**Truxima**, **Rituxan**, **Ruxience**)	Polisorbato 80
	Rituximab and ialuronidasi (**Rituxan Hycela**)	Polisorbato 80
	Temsirolimus (**Torisel**)	Polisorbato 80
	Temozolomide (**Temodar**)	Polisorbato 80
Contraccettivi	Medrossiprogesterone acetato (**Depo-Provera, Depo-Provera CI, Depo-subQ provera 104**)	Polisorbato 80

Corticosteroidi	Metilprednisolone acetato (**Depo-Medrol**)<br>Triamcinolone acetonide (**Aristocort Forte, Aristospan, Kenalog-40, Kenalog-10, Protherix, Triesence, Triloan Suik, Triloan II Suik, Zilretta**)	Polisorbato 80<br>Polisorbato 80
Test Diagnostici	Sincalide (**Kinevac**)<br>Derivato proteico purificato della tubercolina (**Aplisol, Tubersol**)	Polisorbato 20<br>Polisorbato 80
Farmaci antireumatici modificanti la malattia (DMARD)	Anakinra (**Kineret**)<br>Tocilizumab (**Actemra**)	Polisorbato 80<br>Polisorbato 80
Enzimi	Velaglucerasi alfa (**Vpriv**)<br>Imiglucerasi (**Cerezyme**)<br>Taliglucerasi alfa (**Elelyso**)	Polisorbato 20<br>Polisorbato 80<br>Polisorbato 80
Agenti di maturazione eritroide	Luspatercept (**Reblozyl**)	Polisorbato 80
Antidoto dell'inibitore del fattore X attivato	Fattore della coagulazione 10°a (ricombinante),<br>inattivato-zhzo (**Andexxa**)	Polisorbato 80
Gonadotropina	Follitropina (**Menopur**, **Follistim**)	Polisorbato 20
Analoghi del fattore di crescita	Somatotropina (**Nutropin AQ Nuspin 5**)	Polisorbato 20
Fattore di crescita ematopoietici	Eritropoietina (**Retacrit**)<br>Pegfilgrastim (**Fulphila, Neulasta, Nyvepria, Udenyca**)<br>Romiplostim (**Nplate**)<br>Darbepoetin alfa (**Aranesp**)<br>Filgrastim (**Neupogen**, **Nivestym**, **Granix**, **Zarxio**)	Polisorbato 20<br>Polisorbato 20<br>Polisorbato 20<br>Polisorbato 80<br>Polisorbato 80
Agenti contro Epatite B e C	Peginterferon (**Pegasys Pegintron**)	Polisorbato 80
Emostatici	Vitamina K (**Phytonadione**)	Polisorbato 80
Immunoglobuline	Immunoglobuline umane anti-epatite B (**HepaGam B, Nabi-HB**)<br>Immunoglobulina anti-D (Rho) (**WinRho**)	Polisorbato 80<br>Polisorbato 80

Immunomodulatori	Interferon beta-1a (**Avonex, Avonex Pen, Plegridy**)<br>Emapalumab (**Gamifant**)<br>Omalizumab (**Xolair**)	Polisorbato 20<br>Polisorbato 80
Immunosoppressori	Micofenolato mofetile (**Cellcept IV**)	Polisorbato 80
Terapie delle malattie infiammatorie intestinali	Vedolizumab (**Entyvio**)	Polisorbato 80
Inbitori di interleuchine	Sarilumab (**Kevzara**)<br>Dupilumab (**Dupixent**)<br>Mepolizumab (**Nucala**)<br>Secukinumab (**Cosentyx**)<br>Tildrakizumab -asmn (**Ilumya**)	Polisorbato 20<br>Polisorbato 80<br>Polisorbato 80<br>Polisorbato 80<br>Polisorbato 80
Inbitore di Kallikreina	Lanadelumab (**Takhzyro**)	Polisorbato 80
Analogo della leptina	Metreleptin (**Myalept**)	Polisorbato 20
Terapie per la degenerazione maculare	Aflibercept (**Eylea**)<br>Ranibizumab (**Lucentis**)<br>Brolucizumab (**Beovu**)	Polisorbato 20<br>Polisorbato 20<br>Polisorbato 80
Anticorpi Monoclonali	Ocrelizumab (**Ocrevus**)<br>Remdesivir (**Veklury**)<br>Romosozumab (**Evenity**)<br>Teprotumumab (**Tepezza**)<br>Atoltivimab/maftivimab/odesivimab-ebgn (**Inmazeb**)<br>Bamlanivimab<br>Burosumab (**Crysvita**)<br>Canakinumab (**Ilaris**)<br>Casirivimab/Imdevimab<br>Eptinezumab (**Vyepti**)<br>Fremanezumab (**Ajovy**)<br>Inebilizumab (**Uplizna**)	Polisorbato 20<br>Polisorbato 20<br>Polisorbato 20<br>Polisorbato 20<br>Polisorbato 80<br>Polisorbato 80<br>Polisorbato 80<br>Polisorbato 80<br>Polisorbato 80<br>Polisorbato 80<br>Polisorbato 80<br>Polisorbato 80

	Raxibacumab	Polisorbato 80
Terapia per la Sclerosi Multipla	Natalizumab (**Tysabri**)	Polisorbato 80
Miorilassanti	Dantrolene sodico (**Dantrium, Ryanodex**)	Polisorbato 80
Inibitori P-selectina	Crizanlizumab (**Adakveo**)	Polisorbato 80
Inibitori di proprotein convertase subtilisin/kexin type 9 (PCSK9)	Alirocumab (**Praluent**)<br>Evolocumab (**Repatha**)	Polisorbato 20<br>Polisorbato 80
Reumatologici	Belimumab (**Benlysta**)	Polisorbato 80
Trombolitici	Tenecteplase (**Tnkase**)<br>Alteplase (**Cathflo Activase**)<br>Reteplase (**Retavase**)	Polisorbato 20<br>Polisorbato 80<br>Polisorbato 80
Vitamine	Calcitriol (**Calcijex, Rocaltrol**)<br>Doxercalciferol (**Hectorol**)<br>Vitamins A, B1, B2, B6, C, D3, E, K (**Infuvite**)	Polisorbato 20<br>Polisorbato 20<br>Polisorbato 80
